# Perception of Social Odor and Gender-Related Differences Investigated Through the Use of Transfer Entropy and Embodied Medium

**DOI:** 10.3389/fnsys.2021.650528

**Published:** 2021-06-11

**Authors:** Sara Invitto, Soheil Keshmiri, Andrea Mazzatenta, Alberto Grasso, Daniele Romano, Fabio Bona, Masahiro Shiomi, Hidenobu Sumioka, Hiroshi Ishiguro

**Affiliations:** ^1^INSPIRE–Laboratory of Cognitive and Psychophysiological Olfactory Processes, Department of Biological and Environmental Sciences and Technologies, University of Salento, Lecce, Italy; ^2^The Thomas N. Sato BioMEC-X Laboratories, Advanced Telecommunications Research Institute International, Kyoto, Japan; ^3^Neurophysiology, Olfaction and Chemoreception Laboratory, Physiology and Physiopathology Section, Neuroscience, Imaging and Clinical Sciences Department, ‘G. d’Annunzio’ University of Chieti-Pescara, Chieti, Italy; ^4^Department of Psychology and NeuroMi, University of Milano-Bicocca, Milan, Italy; ^5^Department of History, Society and Human Studies, University of Salento, Lecce, Italy; ^6^Interaction Science Laboratories, Advanced Telecommunications Research Institute International, Kyoto, Japan; ^7^Hiroshi Ishiguro Laboratories, Advanced Telecommunications Research Institute International, Kyoto, Japan; ^8^Graduate School of Engineering Science, Osaka University, Osaka, Japan

**Keywords:** social odor, transfer entropy, gender differences, embodied medium, gender voice

## Abstract

The perception of putative pheromones or social odors (PPSO) in humans is a widely debated topic because the published results seem ambiguous. Our research aimed to evaluate how cross-modal processing of PPSO and gender voice can affect the behavioral and psychophysiological states of the subject during a listening task with a bodily contact medium, and how these effects could be gender related. Before the experimental session, three embodied media, were exposed to volatilized estratetraenol (Estr), 5α-androst-16-en-3 α-ol (Andr), and Vaseline oil. The experimental session consisted in listening to a story that were transmitted, with a male or female voice, by the communicative medium via a Bluetooth system during a listening task, recorded through 64-active channel electroencephalography (EEG). The sense of co-presence and social presence, elicited by the medium, showed how the established relationship with the medium was gender dependent and modulated by the PPSO. In particular, Andr induced greater responses related to co-presence. The gender of the participants was related to the co-presence desire, where women imagined higher medium co-presence than men. EEG findings seemed to be more responsive to the PPSO–gender voice interaction, than behavioral results. The mismatch between female PPSO and male voice elicited the greatest cortical flow of information. In the case of the Andr–male voice condition, the trained model appeared to assign more relevance to the flow of information to the right frontotemporal regions (involved in odor recognition memory and social behavior). The Estr–male voice condition showed activation of the bilateral frontoparietal network, which is linked to cognitive control, cognitive flexibility, and auditory consciousness. The model appears to distinguish the dissonance condition linked to Andr matched with a female voice: it highlights a flow of information to the right occipital lobe and to the frontal pole. The PPSO could influence the co-presence judgements and EEG response. The results seem suggest that could be an implicit pattern linked to PPSO-related gender differences and gender voice.

## Introduction

Recent literature about the olfactory system indicates that olfactory perception plays a sensorial role and has implications on social and affective cognition (Herz and Schooler, 2002). The power of olfactory perception to influence social preferences has been highlighted since ancient times: some smells can influence human behavior (Li et al., 2007). During a cross-modal face recognition stimulation (i.e., visual and olfactory stimulation), the behavior of a subject could be conditioned according to the type of olfactory stimulus presented. Li et al. demonstrated that subliminal smells can influence social sympathy judgments and autonomous responses in a consistent manner, and that the behavioral effects on social preferences emerge only when odor information is small enough to prevent top-down regulation. Studies like these have shown that the act of perceiving odors is not merely for its own interest but implies numerous other modifications of a different nature that also cross, obviously, physiological processes (Wacker and Ludwig, 2012). Moreover, interesting research has shown that the smells of the human body can provide important information at the level of social signals (Pause, 2012). This has a peculiar character if we think that social communication could be effectively associated with a very important function given by the result of an underlying process of chemosensory perception in humans (Stevenson, 2009). To explore the above-mentioned aspects, it could be assumed that the processing of human social information should be similar to the processing of chemosensory signals. The elaboration of human social chemosignals resembles the elaboration of social signals from other modes, except that human social chemosignals are usually communicated without the allocation of attentional resources and are thus below the threshold of consciousness (Pause, 2012). In everyday life, humans engage in continuous social relations that affect information processing, both at a conscious level and, as previously observed, under the conscious threshold. Embodied communication involves many of these aspects, which are purely connected to physical contact and the gender dimension. For example, contact is a key component of social interactions because it can mitigate physical and psychological stress (Fisher et al., 1976). Reciprocal contact plays a very important role in governing emotions and physical well-being, and it is the first channel of interaction at the ontological level. The effects of physical contact are peculiar in transmitting emotions and reducing panic (Gallace and Spence, 2010). Moreover, recent studies have shown that mutual contact has a very powerful force in human development because it shapes social reward, attachment, knowledge, communication, and emotional regulation (Cascio et al., 2019). Although we are not yet certain about the possible endocrine changes that they would bring, it is precisely on the basis of the assumptions that, over the years, new communication technologies, have developed an increasing interest in introducing haptic sensations to reproduce the psychophysiological effects induced by physical contact (Field, 2010; Gallace and Spence, 2010). While the psychological and behavioral effects of physical contact, with artificial or robotics systems, have been amply demonstrated, it remains unclear whether the artificial realization of interpersonal contact could produce physiological responses in the same way as they happen in human interactions (Wada et al., 2005; Wada and Shibata, 2006). Researchers have recently shown, however, that communication with a distant person via embracing a physical medium device influences the neuroendocrine system, a phenomenon that could happen in a real situation (Sumioka et al., 2013). The researchers examined cortisol, which is a direct indicator of the effects of psychological, social, and clinical stress (Clow et al., 2004; Ditzen et al., 2007; Mazzatenta et al., 2013; Sumioka et al., 2013). These findings have also been confirmed in terms of electrophysiological activation. Furthermore, numerous studies have been carried out to understand the social odor effect; in particular, some studies indicate that putative pheromones induce different psychophysiological and behavioral responses (Fisher et al., 1976; Doty, 2001; Preti et al., 2003; Johansson and Jones, 2007; Secundo et al., 2014). We can define pheromones as aerial chemical signals that are released into the environment by an individual and influence the psychology and behavior of other subjects of the same species (Karlson and Lüscher, 1959). This type of chemical signal provides information about gender and reproductive status, mediates social and sexual behaviors, and alters neuroendocrine processes (Spehr et al., 2006). The most commonly used putative pheromone substances are estratetraenol (Estr) (Vermetten and Bremner, 2003; Hare et al., 2017; Oren and Shamay-Tsoory, 2019; Ye et al., 2019) and 5α-androst-16-en-3α-ol (Andr) (Morofushi, 2000; Shinohara, 2000; Jahanfar et al., 2007). Pheromone perception in humans is much debated, and researchers have yet to report positive results to detect a conscious perception of pheromones in humans, especially for physical attraction (Black and Biron, 1982). Furthermore, is already demonstrated in the literature, that the pheromonal pathway is independent of the olfactory pathway and projects to the limbic system and therefore does not reach the areas of the conscious olfactory perception, unlike the olfactory pathway, both in mouse models (Dulac and Torello, 2003) and followed by human studies (Savic et al., 2001; Berglund et al., 2006; Savic and Lindström, 2008; Mazzatenta et al., 2013). Moreover, where pure molecules are not used, using the human sweat or a mixture of short-chain fatty acids to investigate also pheromonal physiological modulations in social behavior, the effect of mixed pheromonal inhalation was assessed only in a covert and unconscious way (Cowley and Brooksbank, 1991). Instead, the auditory channel seems to be more sensitive to this gender aspect. Numerous studies have shown that, in the differentiation of gender voices, there is a preference for the female voice; a clear example may be the voices used in video games (Scherer, 1995; Johnson et al., 1999; Kuznekoff and Rose, 2013; Agnew et al., 2018). This female-voice preference has also been confirmed with electroencephalography (EEG) (Titova and Näätänen, 2001). Starting from the above-mentioned literature assumptions and employing methodology, linked to a communication medium, we aimed to evaluate, within a medium relationship (Sumioka et al., 2013; Keshmiri et al., 2018), where the medium doesn’t have any physical characteristic gender related, inserting multisensory-related gender variability (i.e., putative pheromone substances and gender voice), how can be modulated by both cognitive (e.g., co-presence perception) and psychophysiological variables (e.g., electrophysiological activations). Compared to the poorly defined results in the literature (Wyatt, 2020), we expected that there could be little evident variation in the overt perceptive response, and a more sensitive covert electrophysiological response (Lundström and Olsson, 2005; Trotier, 2011). To investigate the effect of androgen/estrogen in information processing of the participants’ cortical activity we choose to use the transfer entropy (TE) analysis (Schreiber, 2000) that aims at extracting directed flow or transfer of information (Lungarella and Sporns, 2006) between interacting processes. TE can also be identified as a conditional mutual information (MI) between two interacting processes (i.e., a causal inference on shared information), even when there are no time-locked events in EEG (King et al., 2013; Di Perri et al., 2016).

## Materials and Methods

This research was conducted at the Laboratory of Cognitive and Psychophysiological Olfactory Processes–INSPIRE LAB–University of Salento (Lecce, Italy). Data collection was carried out in accordance with the Declaration of Helsinki and written informed consent was obtained in advance by all participants. The research protocol was approved by the Ethical Committee of the ASL Vito Fazzi Hospital Lecce–Italy (record number 29, data approval February 11, 2019).

### Subjects

Twenty healthy university students [mean (*M*) = 22.6, standard deviation (*SD*) = 2.4 years] volunteered for this study; they were not compensated. The sample was matched by sex by dividing it into two subgroups (10 men and 10 women, respectively). Sample size was determined a priori according to the relevant related literature as per standard in this field (Pause et al., 1996; Martin, 1998; Anderson et al., 2003; Hummel et al., 2007; Kuang and Zhang, 2014). No subject had a prior history of neurological or psychiatric illness or current or prior psychoactive medication use. In the personal data sheet none of the participants reported any sensorial disease and any an altered olfactory capacity neither due to a chronic condition nor due to an acute condition (e.g., cold, allergic rhinitis or variations connected to temporary aspects) (Hoenen et al., 2016, 2017; Ravia et al., 2020). Participants were asked to abstain from using perfume on the day of recording and to abstain from caffeine and tobacco use for 6 h before testing.

### Stimuli and Task

In this study the “Hugvie” mediums were stored in the laboratory in three different plastic containers (i.e., Box N- Neuter; Box E–Estr, and Box A–Andr), inside which, for each condition, four vials with different substances were inserted, as described below. All the vials were placed with the cap off but were not in contact with the embodied medium’s fabric (Morofushi, 2000; Shinohara, 2000). Before starting the experimental protocol, the Hugvie were stored in the boxes for 2 weeks, at a constant temperature in the laboratory maintained at 21° centigrade. Subsequently, after each use, the Hugvie were always placed in the same boxes marked with the codes N, E, and A. For condition N, the embodied medium was stored in a plastic food box that contained four vials each with a 1 mL suspension of 10 mg Vaseline oil (VO). VO was considered as control substance because it is odorless and doesn’t elicit any significant behavioral, electrophysiological and/or metabolic olfactory response (Invitto et al., 2017; Invitto and Mazzatenta, 2019). In the first condition (Box N–Neuter) the embodied medium N was manipulated by either a male or a female experimenter. For condition E, the Hugvie was stored in a plastic food box that contained four vials each with a 1 mL of 10 mg of pure Estratetraen-3-ol-17-one (Sigma-Aldrich; CAS Number 474-86-2; CAS Number Data Sheet 9.1: Odorless) and 10 mg VO. In the second condition (Box E–Estr), the Hugvie E was manipulated exclusively by a female experimenter. For condition A, the embodied medium was stored in a plastic food box containing four vials of a 1 mL suspension of 10 mg of pure 5α-Androst-16-en-3α-ol (Sigma-Aldrich; CAS Number 1153-51-1; CAS Number Data Sheet 9.1: No Data Available) and 10 mg of VO (Klinkenberg et al., 2011). In the third condition (Box A–Andr), the embodied medium A was manipulated exclusively by a male experimenter. All the experimental session were recorded in the morning from 9.00 to 15.00. The contents of the N, A, and E vials were never changed during the experiment.

### Pheromone Volatilized Compounds Control

To better understand the effect of chemical substances volatilized by the embodied media (EBM) a control experimental assessment was performed under standardized conditions in a well-aired/odorless room, without any bias and with the temperature set at 23°C. The volatilized compounds were measured in the real-time setting using an e-nose sensor (iAQ-2000; Applied Sensor, Warren, NJ, United States) according to a standard analytical method (Mazzatenta et al., 2013, 2015; Invitto and Mazzatenta, 2019). A series of 10 min recordings were conducted in laboratory according to the environmental conditions already described; sensor was placed in an holding support at 5 cm from source: measurements on EBM (baseline), EBM conditioned with VO, Andr, and Estr were conducted in pseudorandom turn. The data analysis was performed using the MatLab, Origin software. To determine the significance of the effects MANOVA and post hoc one-way ANOVA series was used, α = 0.001.

### Narrative Task

**FIGURE 1:**
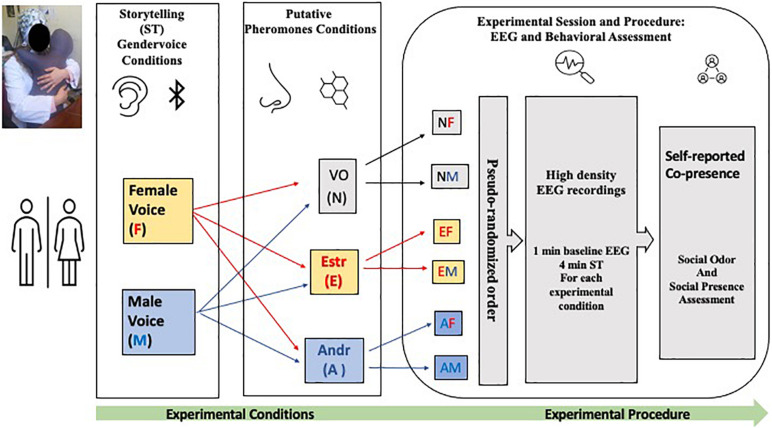
Schematic representation of the experimental conditions and electrophysiological and behavioral assessment. The position of the Hugvie was pseudo randomized for each experimental condition. The duration of each story telling was of 4 min preceded by 1 min of electroencephalography (EEG) baseline recording.

For all three conditions (Neuter, Estr, and Andr), each narrative session of the story was presented alternately in sequence to both the right and the left ears to avoid distortions due to lateralization. Considering the length of each story (4 min) and the repetition for different conditions (six per session), the total task duration was 24 min (see Figure 1). Table 1 and Figure 1 shows these conditions: Andr–female voice (AM), Andr–male voice (AM), Estr–female voice (EF), Estr–male voice (EM), Neuter–female voice (NF), and Neuter–male voice (NM). The listening task lasted 24 min (six conditions of 4 min each). The research task was articulated in two sessions of narration, of which one was narrated by a male voice and the other was narrated by a female voice. The subjects listened to the Italian version of “The Fall of Freddie the Leaf” and “La Foglia Muriel” by Leo F. Buscaglia, which has a duration of 4 min. The reproduction of the story was obtained by inserting a Bluetooth speaker inside a special front pocket on each Hugvie. While listening to the narration, the subject embraced the Hugvie and kept it close for the duration of each session. The experiment began with a 60-s baseline EEG to keep the participant in a neutral emotional state before they began listening to the storytelling. The subjects did not have any kind of information on the differences in conditions/sessions due to the putative pheromone substances. At the end of each session, subjects completed the co-presence and social presence questionnaire (Nowak and Biocca, 2003). At the end of the last experimental session were asked to indicate if they perceived any olfactory variation, through the question “did you perceive an olfactory variation linked to the experimental conditions in the presence of Hugvie”, assessed with a double choice question (the reply could be Yes or No).

**TABLE 1:** Storytelling conditions, narrator’s voice, and duration of the storytelling session.

Condition	Narrator’s gender	Duration (minutes)
Neuter	Male	4
Neuter	Female	4
Estr	Male	4
Estr	Female	4
Andr	Male	4
Andr	Female	4

### Electrophysiological Data Acquisition

Electroencephalography was recorded from the scalp using a 64 active electrode cap (ActiCHamp, Brain Products, Munich, Germany), according to the international 10–10 system, with a sampling frequency of 1,000 Hz. Eye movements were monitored with electrodes attached at the top and the bottom of the left eye and at the top of the right eye. The reference electrode was in FCz and the signal was offline referenced on the mastoid electrodes. Impedance was kept under 5 k. The signal was offline filtered (0.5–50 Hz, 24 dB), and the threshold for artifact rejection was set at 125 μV.

### Overview of TE: Directed Functional Connectivity of the EEG Channel Recordings

We used TE (Schreiber, 2000) to quantify the effect of androgen/estrogen mis/match on information processing of the participants’ cortical activity while listening to the stories that were told by fe/male storytellers through an embodied medium. TE aims at extracting directed flow or transfer of information (Lungarella and Sporns, 2006) between interacting processes. In essence, TE quantifies the deviation from generalized Markov property *p*(*y*_*t* + 1_|*y*_*t*_,*x*_*t*_) = *p*(*y*_*t* + 1_|*y*_*t*_),∀*y*_*t*_,*y*_*t* + 1_ ∈ *Y*,*x*_*t*_ ∈ *X*, where *p*(*y*|*x*) represents the probability of occurrence of *x*, given *y* occurred. As a result, TE computation explicitly answers the question: “how much additional information does the past state of process *X* contain about the future observation of a value of *Y* given that we already know the past state of *Y*?” In this respect, the use of TE for paired EEG channels analysis in the present study is analogous to quantifying how much of each EEG channel’s activity can be explained/understood (in a statistical sense) by observing other EEG channels. More specifically, since TE is explicitly and strictly non-symmetric under exchange of the role of the interacting processes (Kaiser and Schreiber, 2002) its computed value through such pairwise comparison quantifies the directed flow of information from the first to second EEG channel (see Supplementary Materialsfor further details).

We used JIDT for TE computation (Lizier, 2014b). JIDT uses Kraskov-St.gbauer-Grassberger (KSG) algorithm (Kraskov et al., 2004) and its extension for computing TE (Gómez-Herrero et al., 2015). KSG estimation builds on the non-linear and model-free capabilities of kernel estimation with bias correction, thereby resulting in a better data efficiency and accuracy as well as being effectively parameter-free. It is considered to provide best solution for MI, conditional MI, and TE for continuous data (Wibral et al., 2014). It is also crucial to note that KSG algorithm uses (Lizier, 2014a) a dynamically altered kernel width to adjust to the density of samples in the vicinity of any given observation, thereby smoothing out the errors in the PDF estimation. In other words, the embedding dimension is estimated by the algorithm. Further discussion on this matter can be found in (Ragwitz and Kantz, 2002; Jánosi, 2004; Wibral et al., 2014). Prior to TE computation, all EEG channels were detrended.

### Determination of Channels of Interest Based on Computed TEs

**FIGURE 2:**
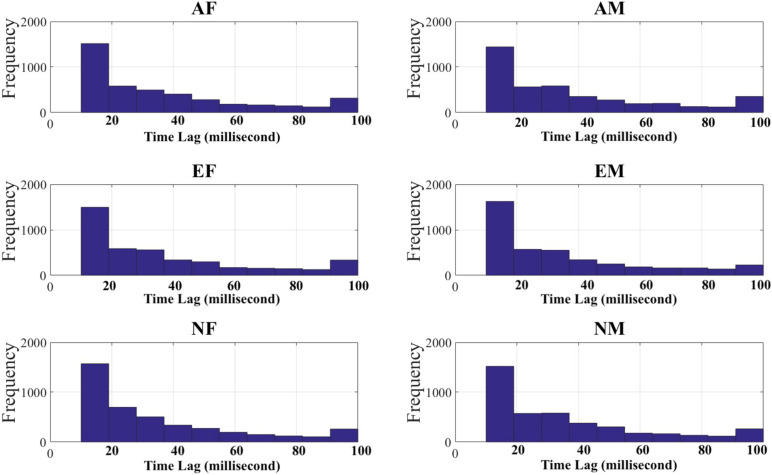
Time lags (in millisecond) associated with maximum transfer entropy (TEs) between every pairs of EEG channels in androgen–female storyteller (AF), androgen–male storyteller (AM), Estr–female voice (EF), estrogen–male storyteller (EM), neuter–female voice (NF), and neuter–male voice (NM) experimental settings.

For each participant in each experimental setting, we first down-sampled their EEG recordings to the tenth of their original length. This resulted in EEG time series of 12,000 data points, per participant, per experimental setting. We then used these down-sampled EEG recordings and computed their paired TEs while optimizing for the choice of time lag that produced the maximum flow of information between them. Specifically, we adapted a brute-force strategy in which we used time lags 0 (i.e., no time lag) through 50 (i.e., equivalent of 500 ms of lag). Wibral demonstrated that, this procedure for computing time lag (i.e., the delay parameter μ in TE equation), results in the maximal TE value (Wibral et al., 2014); this value is identical to the true information transfer delay, between the processes under consideration. We then chose the TE of the time lag that was maximum among all TEs associated with the time lags 0 through 50 (per participant, per EEG channels’ pair, per experimental setting). Figure 2 shows the distribution of time lags for each of the experimental settings. In this figure, it is apparent that most TEs were associated with shorter time lags (Table 2).

**TABLE 2:** Mean (*M*) and standard deviation (SD), and 95.0% confidence interval (CI_95%_) of time lags (in millisecond) associated with paired EEG channels’ computed TEs in different experimental settings.

Setting	*M*	*SD*
AF	3.54	2.88
AM	3.64	2.92
EF	3.56	2.90
EM	3.35	2.77
NF	3.31	2.75
NM	3.43	2.77

*The entries of this table verifies that the transfer entropy (TEs) were associated with short time lag between paired electroencephalography (EEG) channels in all experimental settings and for all pairs of channels.*

Considering the above procedure, there are two points that demand further elaboration. First, computing TE per individual and as per each experimental setting is not new to our study but a common practice. For instance, computing global field potential (Lehmann and Skrandies, 1980; Haenschel et al., 2000), which is based on computing the point-wise root-mean-square-error among EEG channels, also requires its computation to be applied on each individuals’ recordings and experimental settings separately. Second, it appears plausible to suspect the above procedure is susceptible to spurious correlation among EEG channels. However, it is crucial to note that TE is not a measure of “coupling strength.” As noted by Wibral “increasing the interaction strength between two systems may lead to their complete synchronization […] and information cannot be transferred. Hence, TE is zero by definition in this case and thus smaller than in cases with smaller coupling strength and incomplete synchronization” (Wibral et al., 2014). More specifically, TE will be zero for both independent and fully synchronized processes (Kaiser and Schreiber, 2002; Hahs and Pethel, 2013).

After computing TEs, per participant, per experimental setting, we subtracted the TEs of the neutral settings from their respective male and female conditions (e.g., TE_*AM*_ – TE_*NM*_, etc.). As a result of this subtraction step, the TEs of all the other settings (e.g., AF, etc.) quantified the residuals of the information flow that potentially exceeded those of neutral settings. Next, we averaged all participants’ TE matrices (i.e., 62 × 62 matrices, one per pairwise EEG TE computation, per participant, per experimental setting) for AF, AM, EF, and EM settings. This resulted in a single TE matrix of size 62 × 62, per setting, that corresponded to the grand average of TEs for these settings. For each of these averaged TE matrices, we then calculated their 95.0% confidence intervals (CI), per setting, using bootstrap test of significance (10,000 simulation runs). Subsequently, we used the upper-bound of each setting’s CIs as their respective thresholds and discarded all TEs in their grand-averaged matrices that were less than this upper-bound. In essence, this is equivalent to a one-tailed test of significant where the values greater than the average at *p* < 0.025 are accepted. For each setting, we then counted the number of non-zero entries for each channel i.e., number of channels that each EEG channel transferred information to (e.g., number of non-zero entries that corresponded to F3 in EM setting’s TE matrix). Next, we combined these counts and applied bootstrap test (10,000 simulation runs) at 95.0% confidence interval on them to obtain the average number of channels that each EEG channel transferred information to (i.e., their corresponding non-zero TE entries). Subsequently, we discarded all the channels, per setting, whose number of non-zero TE entries were the average number of non-zero entries that was estimated through this step. Last, we found the channels that were common between AF, AM, EF, and EM settings. We found that F3, F2, C2, P5, P6, and PO3 were common between AF, AM, and EM settings. On the other hand, EF did not have any common channels with AF, AM, and EM that passed the TE significance step. Therefore, we discarded EF from our further analyses. For AF, AM, and EM settings, we used their common channels (i.e., F3, F2, C2, P5, P6, and PO3) for comparative analysis of the effect of androgen/estrogen mis/match on information processing of the participants’ cortical activity while listening to the stories that were told by fe/male storytellers through an embodied medium in AM, AF, and EM settings.

## Analysis

### Sensitivity Analysis

Sensitivity analysis leads to estimate the smallest effect size detectable as significant given fixed sample size, alpha error probability and, crucially, power. We set alpha at the standard 0.05, beta at the standard 0.2 (resulting in a power of 0.8), and we fixed the sample size to 20 (our observed sample size). In a repeated measure design, the correlation between the repeated measures has to be fixed too. We conservatively set a value of 0.4 which was a safe estimation based on our observed behavioral data. Notably, the larger the correlation across the repeated measures the more sensitive is the analysis. Because physiological measures tend to correlate more than the behavioral one in the repeated measure designs, we set the sensitivity to the conservative values of the behavioral data. Sensitivity analysis was run with G^∗^Power 3.1 software. The resulting effect size was *f* = 0.258 corresponding to an eta squared = 0.062, a Cohen’s *d* = 0.516, or an *r* = 0.249. These values can be classified as effects of small-to-medium size. More relevantly, they are in the range of the effect that we observed, suggesting that it is unlikely that the study was underpowered. Future studies with a confirmatory scope may adopt our observed effect size to anchor an a priori power analysis.

### Co-presence and Social Presence Analyses

To measure the behavioral response to the experimental protocol, a nine-item questionnaire was administered to the participants after each condition. The questionnaire is a self-report instrument with a 5-point Likert scale (1 = *strongly agree* to 5 = *strongly disagree*). Thus, lower values indicate higher presence (Nowak and Biocca, 2003). The current version was adapted from the original instrument for the specific purposes of this study. Thus, two out of the three original subscales were maintained. Specifically, we kept five items measuring self-reported co-presence (i.e., the feeling of a connection between two actors) and four items measuring social presence (i.e., the perceived ability of the medium to connect the actors). Social presence has default sliding scale from 1 to 100. We changed these four items from a sliding scale to a 5-point Likert scale to make it homologous with the co-presence questionnaire. Thus, for each participant and condition, we had a score for co-presence and a score of social presence; we analyzed these scores independently. We conducted main analyses by adopting analysis of variance (ANOVA) in a general linear model design. The factorial design included the between-subject factor group (men and women) and the within-subjects factors social odor condition (N,E, and A) and voice (*M* and *F*). A lack of sphericity was corrected with the Greenhouse–Geisser method. The *p* values of post hoc tests were corrected for multiple comparisons with the Bonferroni–Holm method. For all statistical tests, the alpha level was set at 0.05.

### Electroencephalography Data

We conducted two types of analyses: (1) test of significant differences among the AF, AM, and EM experimental settings to determine whether the TEs associated with the common channels (i.e., F2, F3, C2, P5, P6, and PO3) in these settings showed any significant difference and (2) the specificity of TEs in AF, AM, and EM settings, in which we utilized the common channels’ TEs of these settings as the input to a logistic regression to determine whether the distribution of information flow from F3, F2, C2, P5, P6, and PO3 (i.e., common channels between AF, AM, and EM) to the remainder of EEG channels was representative of their respective experimental settings. We elaborate on these two analysis steps below.

### Test of Significant Difference Among the Common Channels’ TEs in AF, AM, and EM Experimental Settings

To decide between parametric and non-parametric tests, we first checked whether computed TEs followed a normal distribution. For this purpose, we applied Lilliefors test with Monte Carlo approximation at the 5.0% significance level (i.e., *p* < 0.05). We found that our data did not follow normal distribution. Therefore, we opted for non-parametric tests. First, we applied Kruskal–Wallis test on the combination of common channels (i.e., F2, F3, C2, P5, P6, and PO3) between AF, AM, and EM. We followed this by paired post-hoc Wilcoxon rank sum tests between every pairs of these experimental settings. Next, we performed channel-wise Wilcoxon rank sum tests between every pairs of settings (e.g., Wilcoxon rank sum test on F3 in AF versus AM settings).

### Transfer Entropy Specificity for Differentiating Among the AF, AM, and EM Settings

We used the common channels’ TEs that corresponded to the AF, AM, and EM experimental settings as input to a logistic regression classifier and performed 1,000 simulation classification runs on this data. We used labels 1, 2, and 3 for channels associated with AF, AM, and EM settings, respectively. For each simulation run, we randomly selected a single channel from each AF, AM, and EM setting as a test and used the remaining channels for training the model. This resulted in 15 × 62 and 3 × 62 train and test sets, respectively, per simulation run. We then trained the logistic regression model using the train set and tested its accuracy on a test set (i.e., three randomly selected channels from AF, AM, and EM settings). Given the three-class classification paradigm on a balanced dataset (i.e., six channels per AF, AM, and EM experimental settings), the chance level accuracy was 33.33%. We then used the Wilcoxon signed rank test to determine whether the prediction accuracy of the logistic regression was above the chance level. In addition to the prediction accuracy, we also reported the precision, recall, and F1 score. Next, we used the logistic regression coefficients (i.e., the model’s weights) that were associated with AF, AM, and EM experimental settings and applied the Kruskal–Wallis test on these weights to determine whether the distribution of TEs in these settings played a significant role in distinguishing among these settings. We followed this with paired post hoc Wilcoxon rank sum tests between the model’s weights pertinent to each pair of AF, AM, and EM settings. It is crucial to emphasize that the purpose of the analysis based on logistic regression was not classification problem solving per se. This would have been invalid because we used the grand averages of the participant’s TEs to form the TE representative matrices for each of the AF, AM, and EM settings. Rather, we opted for this analysis to determine whether the distribution of the information flow from the common channels to the other cortical regions bore a significant effect on manifesting the cortical activity that was associated with AF, AM, and EM settings. In other words, we performed this step to further verify how the average (i.e., the expectation) distributed cortical information processing led to quantification of the observed differences that were related to AF, AM, and EM experimental settings.

For Kruskal–Wallis tests we reported the effect size:

$$r=\sqrt{\frac{\chi^{2}}{N}},$$

as suggested by Rosenthal and DiMatteo (2001). In the case of Wilcoxon test, we used (Rosenthal, 1994)

$$r=\frac{W}{\sqrt{N}}$$

as the effect size, with *W* denoting the Wilcoxon statistics. *N* is the sample size in both cases. The effect size in non-parametric tests is considered (Tomczak and Tomczak, 2014) small when *r* ≤ 0.3, medium when 0.3 < *r* < 0.5, and large when *r* ≥ 0.5.

## Results

### Pheromones Volatilized Compounds Results

**FIGURE 3:**
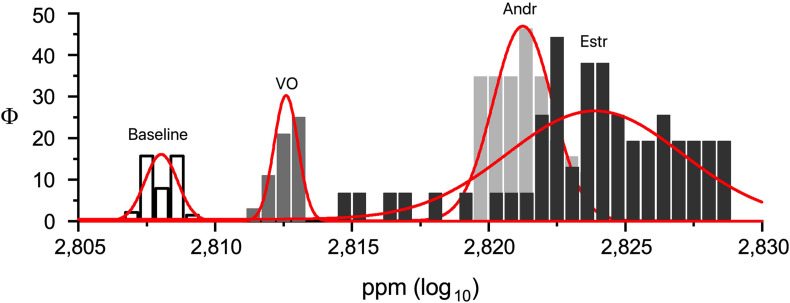
Statistical box and whiskers graphs showing real time of embodied media (EBM) and EBM conditioned with VO, Andr, and Estr recordings.

**FIGURE 4:**
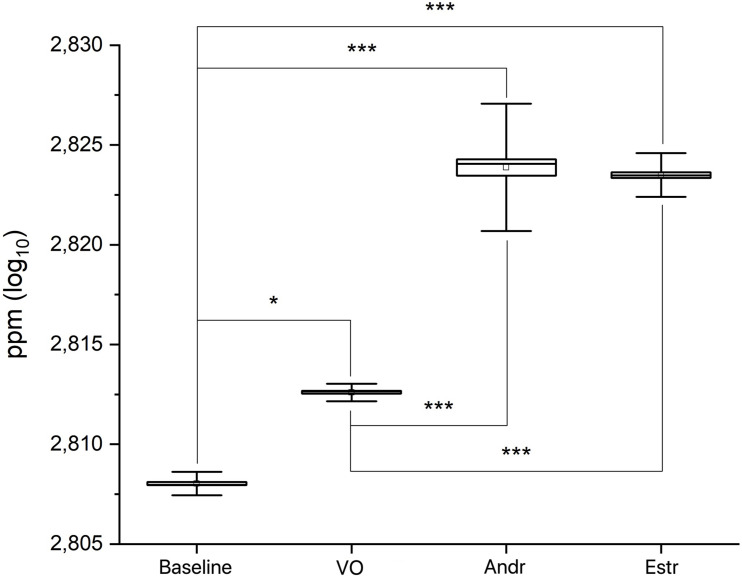
Superimposition of volatile compounds frequency profile recorded, respectively, from EBM and EBM conditioned with VO, Andr, and Estr (^∗^*p* ≤ 0.05; ^∗∗∗^*p* < 0.001).

A preliminary MANOVA return *p* << 0.0001 [*F*_(3,239)_ = 1271.9; mean: baseline 2.808 ± 0.0006 SD; VO 2.813 ± 0.0004 SD; Andr 2.824 ± 0.0032 SD; Estr 2.823 ± 0.001 SD]. A post hoc series of one-way ANOVAs return *p* << 0.0001 for: baseline versus VO [*F*_(1,119)_ = 2302.7]; baseline versus Andr [*F*_(1,119)_ = 1431.7]; baseline versus Estr [*F*_(1,119)_ = 9299.1]; VO versus Andr [*F*_(1,119)_ = 736.8]; VO versus Estr [*F*_(1,119)_ = 5140.3] (see Figure 3). Figure 4 shows a superimposition of volatile compounds frequency profile recorded, respectively, from baseline, VO, Andr, and Estr, respectively; high significant data distribution fit is for baseline *R*^2^ = 0.78, VO *R*^2^ = 0.95, Andr *R*^2^ = 0.75, Estr *R*^2^ = 0.93. According to our results, EBM Baseline control recordings, in comparison with EBM, conditioned with VO, Andr, and Estr, show volatile odorless emission in baseline condition versus control VO and pheromones.

### Self-Reported Co-presence

All the subjects reported no subjective olfactory variation in perception during the three conditions. Therefore, the analysis was not performed for this item due to the absence of any variations reported by the subjects.

The social odor condition [*F*(1.88,31.95) = 4.705, *p* = 0.018, $\rho_{P}^{2}$ = 0.217] and the group [*F*(1,17) = 4.529, *p* = 0.048, $\rho_{P}^{2}$ = 0.210] main effects were significant. However, the voice main effect was not significant [*F*(1,17) = 0.457, *p* = 0.508, $\rho_{P}^{2}$ = 0.026]. None of the interactions were significant. Post hoc tests showed that the social odor condition Andr induced higher co-presence (i.e., lower scores) than the Estr (*t* = 2.895, *p* = 0.020, Cohen’s *d*_*z*_ = 0.66) and Neuter (*t* = 2.326, *p* = 0.052, Cohen’s *d*_*z*_ = 0.53) conditions. There was no difference between the Neuter and Estr conditions (*t* = 0.569, *p* = 0.573, Cohen’s *d*_*z*_ = 0.13). Women had higher co-presence than men (*t* = 2.128, *p* = 0.048, Cohen’s *d*_*z*_ = 0.48).

### Social Presence

The social odor condition [*F*(1.76,29.89) = 0.552, *p* = 0.559, $\rho_{P}^{2}$ = 0.031] and voice [*F*(1,17) = 0.005, *p* = 0.947, $\rho_{P}^{2}$ < 0.001] main effects were not significant. In addition, their interaction was not significant. The group main effect was close to being significant [*F*(1,17) = 3.912, *p* = 0.064, $\rho_{P}^{2}$ = 0.187], and thus we considered the results cautiously. Women had higher social presence (i.e., lower scores) than men (Cohen’s *d*_*z*_ = 0.45).

### Test of Significant Differences Among the Common Channels’ TEs in AF, AM, and EM Settings

**FIGURE 5:**
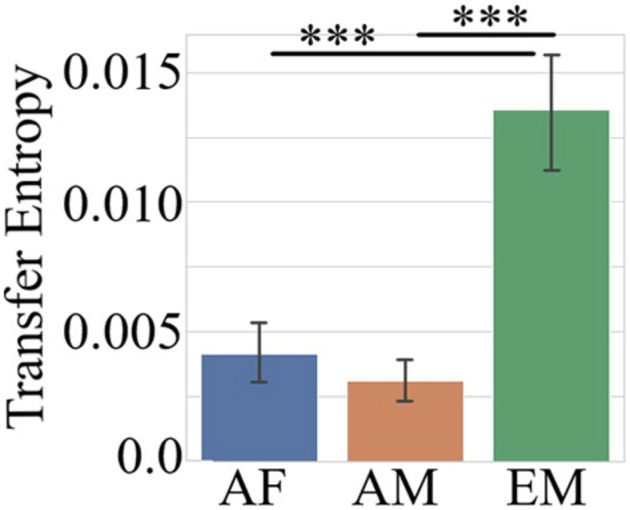
Post hoc Wilcoxon rank sum test on combined TEs of common channels (i.e., F3, F2, C2, P5, P6, and PO3) between AF, AM, and EM settings. The data represent the averaged TEs, per common channel, per setting. In this figure, the asterisks mark significant differences between the settings (^∗∗∗^*p* < 0.001).

The Kruskal–Wallis test indicated that the combined TEs of the common channels (i.e., F2, F3, C2, P5, P6, and PO3) in the AF, AM, and EM settings differed significantly [*H*(2, 1079) = 83.29, *p* = 0.00, *r* = 0.28]. Paired post hoc Wilcoxon rank sum tests (Figure 5 and Table 3) further identified that EM was associated with significantly higher TEs than AF and AM. On the other hand, there was no difference between AF and AM.

**TABLE 3:** Paired Wilcoxon ranksum tests between estrogen–male storyteller (EM), androgen–female storyteller (AF), and androgen–male storyteller (AM).

	*M*	SD	*W* (718)	*p*	*r*
EM versus AF	*EM* = 0.014, *AF* = 0.004	*EM* = 0.02, *AF* = 0.01	–7.99	0.00	–0.30
EM versus AM	*M*_*AM*_ = 0.003	*SD*_*AM*_ = 0.01	–7.48	0.00	–0.28
AF versus AM	–	–	–0.97	0.33	–0.04

*Columns “*M*”, “SD”, “*W*”, “*p*,” and “*r*” are mean, standard deviation, test-statistics, *p*-value, and effect size associated with these tests.*

**FIGURE 6:**
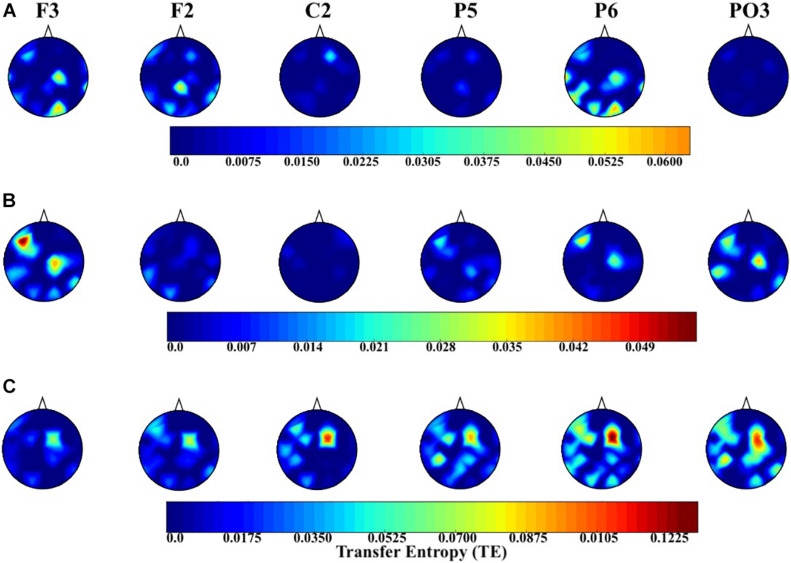
Distribution of information flow from the common channels (i.e., F2, F3, C2, P5, P6, and PO3) to the remainder of channels in the AF (**A** level in the image), AM (**B** level in the image), and EM (**C** level in the image) settings. In this figure, the source (i.e., region from which the information flowed to the other regions) is highlighted at the top of each subplot.

Figure 6 shows the topographic maps of the information flow from common channels (i.e., F2, F3, C2, P5, P6, and PO3) to the remainder of the EEG channels in AF (Figure 5A), AM (Figure 5B), and EM (Figure 5C) experimental settings. There is an apparent increase in flow of information in the case of EM in central (i.e., C2), parietal (i.e., P5 and P6), and parieto-occipital (i.e., PO3) regions. Furthermore, EM’s C2 exhibited an increase in information flow that was lateralized toward the left hemisphere. On the other hand, posterior channels were associated with bilateral information flow. Interestingly, these changes in information flow appeared to highlight the attention network that extends over the frontoparietal regions.

**FIGURE 7:**
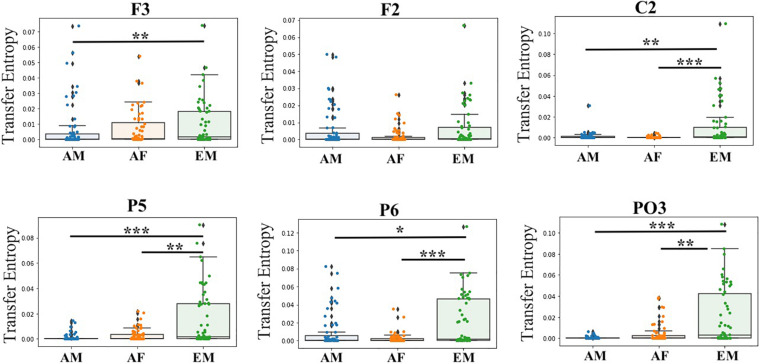
Paired Wilcoxon rank sum test between common channels (i.e., F3, F2, C2, P5, P6, and PO3) in AF, AM, and EM settings. Asterisks mark significant differences in the channels between settings (*p < 0.05; **p < 0.01; ***p < 0.001).

In the case of EM versus AF, pairwise Wilcoxon rank sum test on common channels (Figure 7) identified further significant differences between their common channels’ TEs. Specifically, we observed that (Table 4) EM had larger TEs than AF in channels P5, PO3, P6, and C2. However, EM and AF did not differ with respect to F3 and F2.

**TABLE 4:** Estrogen–male storyteller versus AF.

	EM	AF	*W* (118)	*p*	*r*
P5	*M*_*EM*_ = 0.02, *SD*_*EM*_ = 0.02	*M*_*AF*_ = 0.001, *SD*_*AF*_ = 0.003	–5.58	0.00	0.51
PO3	*M*_*EM*_ = 0.02, *SD*_*EM*_ = 0.03	*M*_*AF*_ = 0.001, *SD*_*AF*_ = 0.001	–5.78	0.00	0.53
P6	*M*_*EM*_ = 0.02, *SD*_*EM*_ = 0.03	*M*_*AF*_ = 0.01, *SD*_*AF*_ = 0.02	–2.43	0.015	0.22
C2	*M*_*EM*_ = 0.01, *SD*_*EM*_ = 0.02	*M*_*AF*_ = 0.001, *SD*_*AF*_ = 0.004	–2.99	0.003	0.27
F3	*M*_*EM*_ = 0.006, *SD*_*AF*_ = 0.01	*M*_*AF*_ = 0.006, *SD*_*AF*_ = 0.01	–0.77	0.439	–0.07
F2	*M*_*EM*_ = 0.01, *SD*_*EM*_ = 0.01	*M*_*AF*_ = 0.007, *SD*_*AF*_ = 0.01	–1.94	0.052	–0.178

*Paired Wilcoxon ranksum tests between common channels (i.e., F2, F3, C2, P5, P6, and PO3). Columns “*W*”, “*p*”, and “*r*” are test-statistics, *p*-value, and effect size associated with these tests. *M* and SD of channels’ TEs are shown under columns EM and AF of this table.*

Similarly (Figure 7), EM (Table 5) had larger TEs than AM in channels P5, PO3, P6, C2, and F3. However, EM and AM did not differ with respect to F2.

**TABLE 5:** Estrogen–male storyteller versus AM.

	EM	AM	*W* (118)	*p*	*r*
P5	*M*_*EM*_ = 0.02, *SD*_*EM*_ = 0.02	*M*_*AM*_ = 0.003, *SD*_*AM*_ = 0.005	–3.20	0.001	–0.29
PO3	*M*_*EM*_ = 0.02, *SD*_*EM*_ = 0.03	*M*_*AM*_ = 0.004, *SD*_*AM*_ = 0.01	–3.11	0.002	–0.28
P6	*M*_*EM*_ = 0.02, *SD*_*EM*_ = 0.03	*M*_*AM*_ = 0.002, *SD*_*AM*_ = 0.01	–4.20	0.00	–0.38
C2	*M*_*EM*_ = 0.01, *SD*_*EM*_ = 0.02	*M*_*AM*_ = 0.0003, *SD*_*AM*_ = 0.001	–4.97	0.00	–0.45
F3	*M*_*EM*_ = 0.006, *SD*_*AF*_ = 0.01	*M*_*AM*_ = 0.002, *SD*_*AM*_ = 0.005	–2.40	0.016	–0.22
F2	*M*_*EM*_ = 0.01, *SD*_*EM*_ = 0.01	*M*_*AM*_ = 0.007, *SD*_*AM*_ = 0.01	–0.81	0.417	–0.07

*Paired Wilcoxon ranksum tests between common channels (i.e., F2, F3, C2, P5, P6, and PO3). Columns “*W*”, “*p*,” and “*r*” are test-statistics, *p*-value, and effect size associated with these tests. *M* and SD of channels’ TEs are shown under columns EM and AF of this table.*

### Transfer Entropy Specificity for Differentiating Among AF, AM, and EM Settings

**FIGURE 8:**
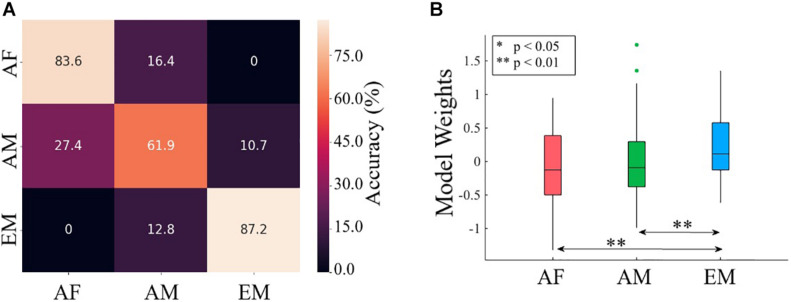
**(A)** Confusion matrix associated with logistic regression classification of common channels (i.e., F2, F3, C2, P5, P6, and PO3) among the AF, AM, and EM settings. **(B)** Paired post hoc Wilcoxon rank sum tests between the logistic regression weights associated with AF, AM, and EM settings. The asterisks mark significant differences between the pairs.

*Note.* These results correspond to 1,000 simulation runs in which three (one per AF, AM, and EM settings) channels were randomly selected (per simulation run) and assigned to the test set and the remaining channels (i.e., 15 channels, five channels per setting) were used for training the logistic regression model. Abbreviations: AF, Andr–female voice; AM, Andr–male voice; EM, Estr–male voice.

Using TEs from common channels to the remainder of channels yielded significantly above chance accuracy (i.e., 33.33% given three classes) in differentiating among AF, AM, and EM settings (Wilcoxon signed rank test: *W*(999) = 27.94, *p* = 0.00, *r* = 0.88). Table 6 provides the mean and standard deviation of the accuracy, precision, recall, and F1-score of 1,000 simulation runs on test set (i.e., three randomly selected channels, one per AF, AM, and EM settings). Figure 8A presents the confusion matrix associated with these results.

**TABLE 6:** Mean (M) and standard (SD) deviation of accuracy, precision, recall, and F1 score of logistic regression classifier for differentiating among AF, AM, and EM settings.

Accuracy (%)	Precision	Recall	F1 score
*M* = 77.57	*M* = 0.69	*M* = 0.78	*M* = 0.71
*SD* = 22.47	*SD* = 0.29	*SD* = 0.22	*SD* = 0.26

**FIGURE 9:**
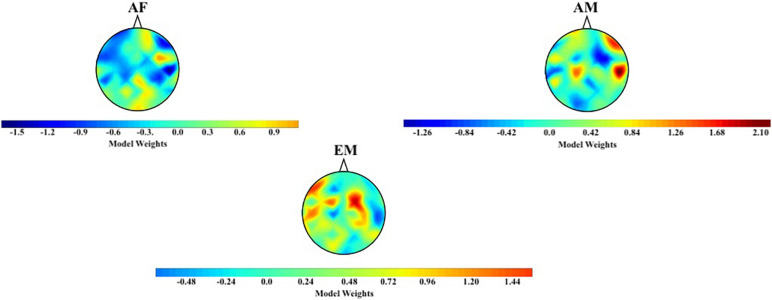
Distribution of logistic regression model’s weights based on TEs from common channels (i.e., F3, F2, C2, P5, P6, and PO3) to the remainder of channels in AF, AM, and EM settings.

Figure 9 shows the topographic maps of the logistic regression model’s coefficients for the AF, AM, and EM experimental settings (Figure 9). These maps revealed that the differences among AF, AM, and EM were not associated with a single and/or small subset of cortical regions and that the overall distribution of the information flow from these common channels (i.e., F3, F2, C2, P5, P6, and PO3) to the remainder of the cortical regions played a role in their observed differences. In the case of AM, the trained model appeared to assign more importance to the flow of information to the right frontotemporal regions. On the other hand, it considers the information flow to the bilateral frontoparietal attention networks (extending to the right-hemisphere temporal region) to be of greater importance while evaluating the EM setting. Finally, the model appears to distinguish AF based on the flow of information to the right-hemisphere occipital and frontal pole.

The Kruskal–Wallis test indicated a significant difference in the model’s weights associated with AF, AM, and EM settings [*H*(2, 179) = 10.71, *p* = 0.005, *r* = 0.24]. Post hoc Wilcoxon rank sum test (Figure 8B) further revealed that the weights associated with EM were higher than AF (*M*_*AF*_ = −0.09, *SD*_*AF*_ = 0.56 and *M*_*EM*_ = 0.21, *SD*_*EM*_ = 0.47; *W*(118) = −2.95, *p* = 0.003, *r* = −0.27) and AM (*M*_*AM*_ = −0.03, *SD*_*AM*_ = 0.60; *W*(118) = −2.67, *p* = 0.008, *r* = −0.24). On the other hand, this test identified a no difference between AF and AM weights [*W*(118) = −0.40, *p* = 0.691, *r* = −0.04].

## Discussion and Concluding Remarks

The human pheromones perception is strongly questioned by recent research, which sees the published data on this topic as an effect of false positives (Wyatt, 2020). Furthermore, human vomeronasal cavities are present when observed through endoscopy appear to be not functional to pheromones perception. Nevertheless, several steroids are considered to be putative human pheromones; some of whose activate the anterior hypothalamus, although this activation might not necessarily related so much to the vomeronasal system but to the olfactory system (Savic et al., 2001). So far, in most studies on pheromones, the response is not a conscious behavioral or perceptual response, but a neuroendocrine or subcortical/emotional activation response (Lundström et al., 2003; Bensafi, 2004; Lundström and Olsson, 2005; Hummer and McClintock, 2009). In this study, starting from recent assumptions both from research in the field of cognitive neuro-olfactometry and new communication technologies, we evaluated if, and if so how, there could be an effect due to the presence of putative pheromone substances linked to the male and female gender. During the methodological assessment of volatile compounds, we found that, as reported in literature (Mazzatenta et al., 2010, 2016) pheromones volatilized and effect on physiology of human subjects, human pheromones typically do not elicit olfactory system but the accessory one affecting limbic system while not affect, in an overt way, the perception/cognition. We also evaluated if this effect could be superimposable or implementable to that of the gender voice of the communicative medium, both at the behavioral and the electrophysiological level. We found the main results with Andr, which is the most important endogenous steroid and is present in some areas of the male body, such as hair and the underarm skin surface. We hypothesize that this putative pheromone substance activates cortical areas related to social cognition and attention compared with non-pheromonic odors. Referring to previous studies that used non-olfactory stimuli for social cognition tests, we expected pheromone-related cortical activation in the lateral and medial prefrontal cortex, in the superior temporal cortex (Frith and Frith, 2006). We found both behavioral and electrophysiological changes in response to the type of protocol presented. In particular, for the social odor and the co-presence component, we found that the social odor, in particular, Andr can predict higher responses related to co-presence. Furthermore, the participants’ gender is related to the co-presence desire, where women imagined greater medium co-presence than men. This result could be connected to greater activation predicted by Andr, the most arousing putative pheromone (Cowley and Brooksbank, 1991; Morofushi, 2000) Even in the social presence dimension, women seem to be more responsive than men. In other words, the manipulation of the conditions appears to be able to predict the outcome, although this does not necessarily imply that they are the cause of the outcome. The potential role of mediator variables might be tested in future studies. Electrophysiological but not behavioral results were responsive to the pheromone–gender voice interaction. However, it is the mismatch between female social odor and male voice that seems to elicit the most cortical flow of information. In the case of the AM condition, the trained model appeared to assign more relevance to the flow of information to the right frontotemporal regions. This cortical stream is strongly involved in odor recognition memory and in social behavior (Jones-Gotman and Zatorre, 1993; Rolls, 2000, 2004; Blood and Zatorre, 2001). On the other hand, it considers the information flow to the bilateral frontoparietal networks (extending to the right-hemisphere temporal region) to be of greater importance while evaluating Estr with a gender voice mismatch. The bilateral frontoparietal network is linked to cognitive control, cognitive flexibility, and auditory consciousness (He et al., 2007; Brancucci et al., 2016; Marek and Dosenbach, 2018). This is in line with the cognitive dissonance that the subjects experience during the narrative medium task. Finally, the model appears to distinguish the other dissonance condition linked to Andr matched with a female voice: this condition highlights a flow of information to the right occipital lobe and to the frontal pole. This cortical pathway is linked to relational processing (Hartogsveld et al., 2018). In this case, a sort of emotional/cognitive disengagement moment is highlighted when the social odor is reversed with the gender of the voice (i.e., female social odor versus male voice). We conclude that putative pheromone substances can be perceived both at the implicit behavioral level and at the electrophysiological level. In fact, subliminal social odors can influence the co-presence judgements and electrophysiological responses in a consistent manner, and it seems that there is an implicit pattern that recognizes social odor–related gender differences and gender–gender (Li et al., 2007). This pattern seems to increase the posterior to anterior cortical flow of information when there is an emotional/cognitive gender mismatch. We conclude that in a complex system, such as that of advanced technological communication through artificial media, the embodied multisensory component can certainly also involve gender-specific and relational aspects, which can be implemented through substances that modulate social odor. In fact, cross modal processing elaborates various sensory levels, perceives the connection of these levels, and is activated when these levels do not follow cognitive expectations, as if there were emotional and communicative scripts also connected to the gender-related social odor. These aspects should be carefully considered in the future, both for advanced technological implementation, including neuromorphic ones, and to understand new aspects of social odor, to have a methodologically controlled model.

## Limitation and Future Direction

The study has some methodological limitation (both to the methodological design and for the statistical design). In fact, this was not a double-blind, but a single-blind study. Only the subjects that participated to the study were blinded on the information related to the use of three different substances than on the three Hugvie. The variation perceived by the subject, with respect to the different conditions, was related to the variation of the narrator’s voice, and nothing else appeared to be changed in the presence of the embodied medium. The embodied mediums were identical, of the same color, and no significant variation in subject’s perception of smell was evident. However, the medium was not handled by male/female experimenter if it belonged to the Estr/Andr. On the other hand, in Neutral case, it could be handled by both the male and the female experimenters. This could be a co-occurring effect (i.e., the gender experimenter’s effect) (Lundström and Olsson, 2005) and could be a limitation of the work. In future studies, only the effect of the experimenter’s gender, in inducing a behavioral or cortical response effect could be investigated, analyzing it in a double blind condition and in a single blind condition. A final consideration could be related to the nature of this study. This study has not the aim to assess an explicit perceptive measurement of substance. According to the model linked to the implicit investigation of covert electrophysiological perception, there are also important studies carried out on subjects where the overt response cannot be measured (e.g., sleep, coma or subthreshold attentive state), which evaluate the electroencephalographic response, also in some cases with the TE, to understand aspects indirectly connected to consciousness and perception (Fernandez-Duque and Posner, 1997; Van Overwalle and Baetens, 2009; Gosseries et al., 2011; Rodríguez-Sotelo et al., 2014; Miskovic et al., 2019). Other relevant studies on consciousness do not evaluate overt aspects but psychophysiological covert aspects through the model of complexity (Bayne et al., 2020). Where perceptual aspects can be highlighted by electrophysiological covert characteristics, it is not necessary to have direct perceptual/behavioral overt comparisons. In any case, in our research study, these behavioral aspects are evaluated through the model of co-presence, which is the model that comes closest to the relational aspects that we were interested in observing by behavioral view.

This study is not intended to overlap with other research fields (e.g., physiology, chemistry, or in the medical evaluation aspects to the research field of ENTs) investigating the metric aspects of the quantity of odorant produced by some substances or perceived by the subjects. Instead, our psychophysiological study, had the aim to investigate the variation in the response of pheromones-like substances, not so much according to olfactometric aspects, but in its connection with gender (narrator’s voice) and the sense of co-presence (in a single-blind condition).

## Data Availability Statement

The data associated with the present study are available on request to the corresponding authors (SI, sara.invitto@unisalento.it or SK, soheil.keshmiri@oist.jp).

## Ethics Statement

The data collection was carried out in accordance with the Declaration of Helsinki and written informed consent was obtained in advance by all participants. The research protocol was approved by the Ethical Committee of the ASL Vito Fazzi Hospital Lecce – Italy (record number 29, data approval February 11, 2019).

## Author Contributions

SI designed and conducted the experiments. SK proposed the use of TE and carried out the analyses. AM performed the volatile compound analysis. DR performed the co-presence and social presence analyses. FB prepared the chemical vials. SI and SK wrote the original and the revised versions of the manuscript. AG took part in data acquisition. MS, HS, and HI contributed to the manuscript preparation and writing. All authors contributed to the article and approved the submitted version.

## Conflict of Interest

The authors declare that the research was conducted in the absence of any commercial or financial relationships that could be construed as a potential conflict of interest.
