# Supplementary material for: Perception of Social Odor and Gender-Related Differences Investigated Through the Use of Transfer Entropy and Embodied Medium
Source: Front Syst Neurosci. 2021 Jun 11;15:650528. doi: 10.3389/fnsys.2021.650528 (PMC8232750; doi:10.3389/fnsys.2021.650528)
Supplement: Supplementary file 1 [file Data_Sheet_1.DOCX]

**Supplementary Materials**

**S.1. Further Background on Transfer Entropy (TE)**

TE is expressed as a specific version of Kullback-Leibler divergence [1] i.e., the relative entropy [2]:

$$TE\left( X\to Y, \mu\right)=\sum_{y_{t}, y_{t-1}^{dy},x_{t-\mu}^{dx}} p(y_{t},y_{t-1}^{dy},x_{t-\mu}^{dx})log\frac{p(y_{t}|y_{t-1}^{dy},x_{t-\mu}^{dx})}{p(y_{t}|y_{t-1}^{dy})}$$

where parameter $\mu$ (referred to as delay embedding i.e., the lagged history) is the assumed time that the information transfer needs to get from X to Y and $y_{t-1}^{dy}$ represents the past of Y. Similarly, $X_{t-\mu}^{dx}$ refers to the past of X while incorporating the delay embedding $\mu$ (ibid). This equation quantifies the degree to which the history of X predicts the current state of Y beyond the degree to which Y could be predicted by its own history, or equivalently [3]:

$$TE\left( X\to Y \right)\equiv H\left( Y_{n} | Y_{n}^{\mu} \right)-H\left( Y_{n} | Y_{n}^{\mu},X_{n}^{\mu} \right)$$

where $H\left( Y_{n} | Y_{n}^{\mu} \right)$and $H(Y_{n}|Y_{n}^{\mu},X_{n}^{\mu})$ give conditional entropy of Y at time n given its $\mu$-lag history and conditional entropy of Y at time n given its own and X’s $\mu$-lag history.

In essence, TE quantifies the deviation from generalized Markov property ${p(y}_{t+1}\left| y_{t},x_{t} \right)=p\left( y_{t+1} | y_{t} \right),\forall y_{t},y_{t+1}\in Y,x_{t}\in X$, where $p(y|x)$ represents the probability of occurrence of $x$, given $y$ occurred. If this deviation is small, then the state of $X$ is assumed to have minimal or no relevance on the transition probabilities of $Y$ [4], thereby implying an absence and/or a non-significant effect of $X$ on $Y$.

It is worthy of note that unlike MI that measures correlation (i.e., a measure of synchrony while taking into account the linear and nonlinear relations), TE is explicitly and strictly non-symmetric under exchange of the role of the interacting processes [5]. In other words, $TE\left( X\to Y \right)\neq TE\left( Y\to X \right),\forall X,Y$.

1. Cover, T.M.; Thomas, J.A. *Elements of Information Theory*; John Wiley and Sons, 2005; ISBN 9780471241959.

2. Wibral, M.; Vicente, R.; Lindner, M. Transfer entropy in neuroscience. *Underst. Complex Syst.* **2014**, 3–36, doi:10.1007/978-3-642-54474-3_1.

3. Barnett, L.; Bossomaier, T. Transfer entropy as a log-likelihood ratio. *Phys. Rev. Lett.* **2012**, *109*, doi:10.1103/PhysRevLett.109.138105.

4. Lungarella, M.; Sporns, O. Mapping information flow in sensorimotor networks. *PLoS Comput. Biol.* **2006**, *2*, 1301–1312, doi:10.1371/journal.pcbi.0020144.

5. Kaiser, A.; Schreiber, T. Information transfer in continuous processes. *Phys. D Nonlinear Phenom.* **2002**, *166*, 43–62, doi:10.1016/S0167-2789(02)00432-3.
